# Role of hepatocyte nuclear factor 4 alpha in cell proliferation and gemcitabine resistance in pancreatic adenocarcinoma

**DOI:** 10.1186/s12935-019-0767-4

**Published:** 2019-03-04

**Authors:** Qiqing Sun, Wenyan Xu, Shunrong Ji, Yi Qin, Wensheng Liu, Qiangsheng Hu, Zheng Zhang, Mengqi Liu, Xianjun Yu, Xiaowu Xu

**Affiliations:** 1 Department of Pancreatic Surgery, Fudan University Shanghai Cancer Center, Shanghai, 200032 China; 2 Department of Oncology, Shanghai Medical College, Fudan University, Shanghai, 200032 China; 3 Pancreatic Cancer Institute, Fudan University, Shanghai, 200032 China; 4 Shanghai Pancreatic Cancer Institute, Shanghai, 200032 China

**Keywords:** Pancreatic adenocarcinoma, Hepatocyte nuclear factor 4α, Human equilibrative nucleoside transporter 1, Proliferation, Gemcitabine resistance

## Abstract

**Background:**

Hepatocyte nuclear factor 4α (HNF4α) is a tissue-specific transcription factor that regulates the expression of numerous genes in hepatocytes and pancreatic β cells. HNF4α has been reported to affect cell proliferation and chemoresistance in several cancers. However, the role of HNF4α in pancreatic adenocarcinoma (PDAC) has not been studied extensively and remains unclear.

**Methods:**

By utilizing immunohistochemical (IHC) staining, we measured the expression of HNF4α in PDAC tissues. By silencing HNF4α in PDAC cell lines, we assessed the impact of HNF4α on pancreatic cancer cell proliferation and gemcitabine sensitivity. We used CCK8 and colony formation assays to examine the effect of HNF4α on cell proliferation. A flow cytometry assay was used to assess cell apoptosis. The expression of gemcitabine-related genes was detected by quantitative real‑time PCR (qRT-PCR) and Western blotting. IHC was utilized to assess the correlation between HNF4α and human equilibrative nucleoside transporter 1 (hENT1) expression in PDAC patients. Chromatin immunoprecipitation (ChIP) and dual‑luciferase reporter assays were used to confirm that hENT1 is a target gene of HNF4α.

**Results:**

Increased HNF4α expression was detected in PDAC tissues; patients with higher HNF4α expression displayed worse prognosis. To elucidate the function of HNF4α, we examined its role in pancreatic cancer cell proliferation, apoptosis and gemcitabine resistance. In HNF4α-silenced Capan-1 and MiaPaCa-2 cells, we observed decreased cell proliferation and increased sensitivity to gemcitabine compared to those of controls. The mechanism of HNF4α in gemcitabine-related chemosensitivity was then explored. In response to HNF4α silencing, the expression levels of gemcitabine-related proteins, hENT1 and deoxycytidine kinase (dCK) were significantly increased. Additionally, hENT1 was negatively correlated with HNF4α in PDAC tissue samples. Moreover, we identified hENT1 as a downstream target of HNF4α.

**Conclusion:**

HNF4α is a prognostic marker for overall survival, is required for pancreatic cancer cell proliferation and promotes resistance to gemcitabine by downregulating hENT1. Therefore, targeting HNF4α might reverse gemcitabine resistance and provide novel treatment strategies for PDAC.

**Electronic supplementary material:**

The online version of this article (10.1186/s12935-019-0767-4) contains supplementary material, which is available to authorized users.

## Background

Due to the lack of early diagnosis and limited therapy options, pancreatic ductal adenocarcinoma (PDAC) is still among the most lethal tumors and is the fourth leading cause of cancer-related deaths in the United States [1, 2]. Despite enormous progress in the treatment of many other tumor types in recent years, the main treatment for PDAC, other than surgery, is still based on gemcitabine alone or in combination with other chemotherapeutic agents [3]. However, the effect of gemcitabine and other chemotherapeutics remains unsatisfactory, partly due to the intrinsic chemoresistant characteristics of pancreatic cancer cells [4]. Therefore, exploration of potential targets to overcome gemcitabine resistance, which may highlight promising directions for developing novel therapeutic strategies, are urgently needed.

Hepatocyte nuclear factor 4α (HNF4α or NR2A1), is an orphan nuclear receptor that is expressed in pancreas, liver, kidney and intestine [5, 6]. HNF4α has been widely studied as an important regulator of hepatic functions, such as glycometabolism, fatty acid metabolism, and drug metabolism [7, 8]. Although there is abundant evidence that HNF4α plays an important role in hepatic development, its role in the regulation of tumorigenesis and cancer development has been less widely studied. Darsigny et al. reported that HNF4α was highly expressed in colorectal cancer tissue samples and promoted murine tumor development by targeting redox-related genes [9]. Conversely, another study suggested that low HNF4α expression in colorectal cancer reduced the expression of the tumor suppressor gene CDX2 and that the lack of HNF4α function promoted tumor progression [10]. Moreover, a recent study has suggested a role for HNF4α in chemoresistance in gastric cancer, in which they reported that HNF4α may enhance multidrug resistance by regulating cell apoptosis and expression of B-cell lymphoma 2 (Bcl-2) [11]. However, the role of HNF4α in PDAC development has not been reported widely and needs further investigation.

As a highly hydrophilic chemical, gemcitabine requires integral membrane transporters to function as a mediator for drug intake. It has been reported that a membrane transporter protein, human equilibrative nucleoside transporter 1 (hENT1), is responsible for the majority of gemcitabine uptake [12, 13]. Once it is in the cytoplasm, gemcitabine undergoes rate-limiting steps, as the monophosphate is phosphorylated by deoxycytidine kinase (dCK) to generate its active metabolites, gemcitabine diphosphate and gemcitabine triphosphate [14, 15]. The most important mechanism of gemcitabine cytotoxicity is inhibition of DNA synthesis, in which ribonucleotide reductase (RR) plays an important role [16–18]. First, the diphosphate form of gemcitabine can inactivate RR subunit M1 (RRM1) and RR subunit M2 (RRM2). Additionally, gemcitabine triphosphate can embed into DNA chains, which causes termination of DNA synthesis, resulting in cancer cell apoptosis [19]. In brief, hENT1, dCK and RR are central proteins that contribute to gemcitabine cytotoxicity, and many previous studies have confirmed that their expression correlates with gemcitabine efficacy and patient prognosis [13, 20, 21].

In the present study, we explored the function of HNF4α and its underlying mechanism in PDAC. Our study provided novel findings, including that HNF4α silencing resulted in decreased cell proliferation and increased gemcitabine sensitivity, which was partly due to transcriptional repression of hENT1. Collectively, our present study uncovered novel predictive and treatment targets that show promise for improving overall survival for PDAC.

## Methods

### Clinical samples and cell lines

Clinical tissue samples were obtained from patients who were diagnosed at the Department of Pancreatic Surgery at Fudan University Shanghai Cancer Center (FUSCC) in 2012. We obtained informed consent from the patients and were approved by the Institutional Research Ethics Committee of FUSCC. The clinical characteristics of the samples are summarized in Table 1. The human pancreatic cancer cell lines Capan-1 and MiaPaCa-2 were purchased from the American Type Culture Collection (ATCC, USA) and were cultured according to standard ATCC protocols. In brief, PANC-1 cells were cultured in Iscove’s modified Dulbecco’s medium containing 20% fetal bovine serum (FBS), 100 U/mL penicillin and 0.1 mg/mL streptomycin. MiaPaCa-2 cells were maintained in Dulbecco’s modified Eagle’s medium with 10% FBS and an additional 2.5% horse serum. These cells were cultured at 37 °C in a humidified incubator with 5% CO_2_.

**Table 1:** Relationship between HNF4α expression and clinicopathological features of pancreatic cancer

Variables	Patient number (n)	HNF4α expression in tumor tissue of pancreatic cancer		
		Low (n = 41)	High (n = 64)	*P* value
Gender				0.9321
Male	62	24	38	
Female	43	17	26	
Age (years)				0.2434
≤ 60	54	24	30	
> 60	51	17	34	
TNM stage				0.8487
I, IIa	55	21	34	
IIb, III, IV	50	20	30	
Tumor size (cm)				0.2764
≤ 4	73	26	47	
> 4	32	15	17	
Histological grade				0.1917
Grade 1, 2	62	21	41	
Grade 3	43	20	23	
Lymph node status				0.8625
Negative	60	23	37	
Positive	45	18	27	
Vascular emboli				0.6061
Negative	87	33	54	
Positive	18	8	10	

### Immunohistochemical (IHC) staining

IHC staining of paraffin-embedded tissues with antibodies against HNF4α (1:200; CST, USA), hENT1 (1:200; Proteintech, USA) and dCK (1:800; Abcam, UK) were performed using standard procedures as described previously [22–24]. The evaluation criterion considered both the percentage of stained positive cells (0, < 5%; 1, 5–25%; 2, 25–50%; 3, 50–75%; 4, > 75%) and the staining intensity (0, negative; 1, weakly positive; 2, moderately positive; 3, strongly positive). The total scores were calculated as the product of staining frequency and intensity. The expression levels were classified as follows: negative (0-3, −); weakly positive (4, +); moderately positive (6, ++); and strongly positive (> 6, +++). The patients were divided into two groups (∓, low expression and ++/+++, high expression) for survival analyses.

### Plasmids

The 21 base pair (bp) targets against HNF4α were TCAGGGTCTGAGCCCTATAAG and CCATCACCAAGCAGGAAGTTA. shRNA oligos were synthesized and ligated into an LKO.1 TRC cloning vector (Addgene, USA) according to the standard procedures provided by Addgene [25], and HNF4α silencing lentivirus constructs were designated pLKO.1-shHNF4α-A and pLKO.1-shHNF4α-B. pLKO.1-shHNF4α was cotransfected with psPAX2 and pMD2.G into HEK-293T cells at a ratio of 4:3:1 for the production of lentiviral particles. pLKO.1-shscramble (Addgene, USA) was used as the control plasmid.

### Quantitative real-time PCR (qRT-PCR)

TRIzol reagent (Invitrogen, USA) was used to extract total RNA. Reverse transcription was performed using a TaKaRa PrimeScript RT reagent kit (TaKaRa, Japan) to obtain cDNA. The expression of candidate genes was determined using an ABI 7900HT Real-time PCR system (Applied Biosystems, USA). The primer sequences used in this study are presented in Table 2.

**Table 2:** Primer sequences used in this study

*HNF4α* forward	5′-GGTGTTCAAGGACGTGCTGCTCC-3′
*HNF4α* reverse	5′-AGTCCTCCAAGCTCACCTGCACC-3′
*hENT1* forward	5′-CTCCAACTCTCAGCCCACCAATGA-3′
*hENT1* reverse	5′-GAAGTAACGTTCCCAGGTGCTGC-3′
*dCK* forward	5′-CAAGACTGGCATGACTGGATGAA-3′
*dCK* reverse	5′-GGCACCTCTTGAAGATAATCGAAG-3′
*RRM1* forward	5′-TGGAGTACACCAGCAAAGATGAGG-3′
*RRM1* reverse	5′-GGCGATGGCGTTTATTTGATAGGC-3′
*β*-*actin* forward	5′-AGAGCTACGAGCTGCCTGAC-3′
*β*-*actin* reverse	5′-AGCACTGTGTTGGCGTACAG-3′
ChIP forward 1	5′-TGCCTCACTGGCCTCTCCCTAGTC-3′
ChIP reverse 1	5′-CACCACCCTATATGGGACCGTGGC-3′
ChIP forward 2	5′-TTTGAATGTGCCCCGGCGGGAGA-3′
ChIP reverse 2	5′-TCCCTGGCCCGTGCGCGCCACGT-3′

### Western blotting analysis

Western blotting was conducted as described in a previous study [24]. The antibodies used in the present study were β-actin (1:5000; Proteintech, USA), HNF4α (1:1000; CST, USA), p21 (1:1000; Proteintech, USA), p27 (1:1000; Proteintech, USA), Caspase-9 (1:1000; CST, USA), PARP (1:1000; CST, USA), ENT1 (1:1000; Proteintech, USA), DCK (1:1000; Abcam, UK), and RRM1 (1:1000; Proteintech, USA).

### CCK-8 assay

Cell proliferation and cytotoxicity was measured via cell viability using a Cell Counting Kit-8 (Dojindo, Japan) and was conducted as previously described [26]. The concentration of gemcitabine that inhibited cell viability by 50% (IC50 value) was calculated from a nonlinear least squares curve that was fit to the dose–response curves.

### Colony formation assay

Cells (500/well) were seeded and cultured in six-well plates, and cells were fixed with 4% paraformaldehyde followed by 0.1% crystal violet (Sigma, USA) staining after 2 weeks of cultivation. Colonies were quantified under a light microscope.

### Cell apoptosis analysis

Cells were stained with fluorescein isothiocyanate-conjugated annexin V and propidium iodide (BD, La Jolla, CA, USA) according to the manufacturer’s instructions, and the percentage of apoptotic cells was measured using a FACSCalibur flow cytometer.

### Promoter activity assessment by dual-luciferase assay

The hENT1 promoter region, spanning from − 3000 to 300 of the transcription starting site, was amplified from genomic DNA and cloned into a pGL3-Basic vector. HEK-293T cells were seeded on 96-well culture plates and transfected with the pGL3 constructs as well as Renilla luciferase expression vectors using Lipofectamine 2000 (Invitrogen, USA). Both firefly and Renilla luciferase activities were assayed using a dual-luciferase system (Promega, USA) that complied with the manufacturer’s protocol.

### Chromatin immunoprecipitation (ChIP) assay

ChIP was conducted according to the instructions of a Magna ChIP™ A/G Chromatin Immunoprecipitation Kit (Merck Millipore Corporation). The nuclear DNA extracts were amplified using two pairs of primers that spanned the hENT1 promoter region (Table 2).

### Statistics

All data are presented as the mean ± SD, and experiments were repeated at least three times. The data were analyzed using SPSS 22.0 software (Abbott Laboratories, USA). Student’s test and one-way ANOVA were used to analyze the data between groups. A Chi square test was performed to analyze the relationships between HNF4α expression and clinicopathologic characteristics. Log-rank test and Cox regression were used in survival analysis for patients with PDAC. Spearman correlation analysis was used to determine the correlation between the HNF4α and hENT1 expression levels. *P* < 0.05 was considered statistically significant.

## Results

### HNF4α expression is positively correlated with PDAC prognosis

**Fig. 1:**
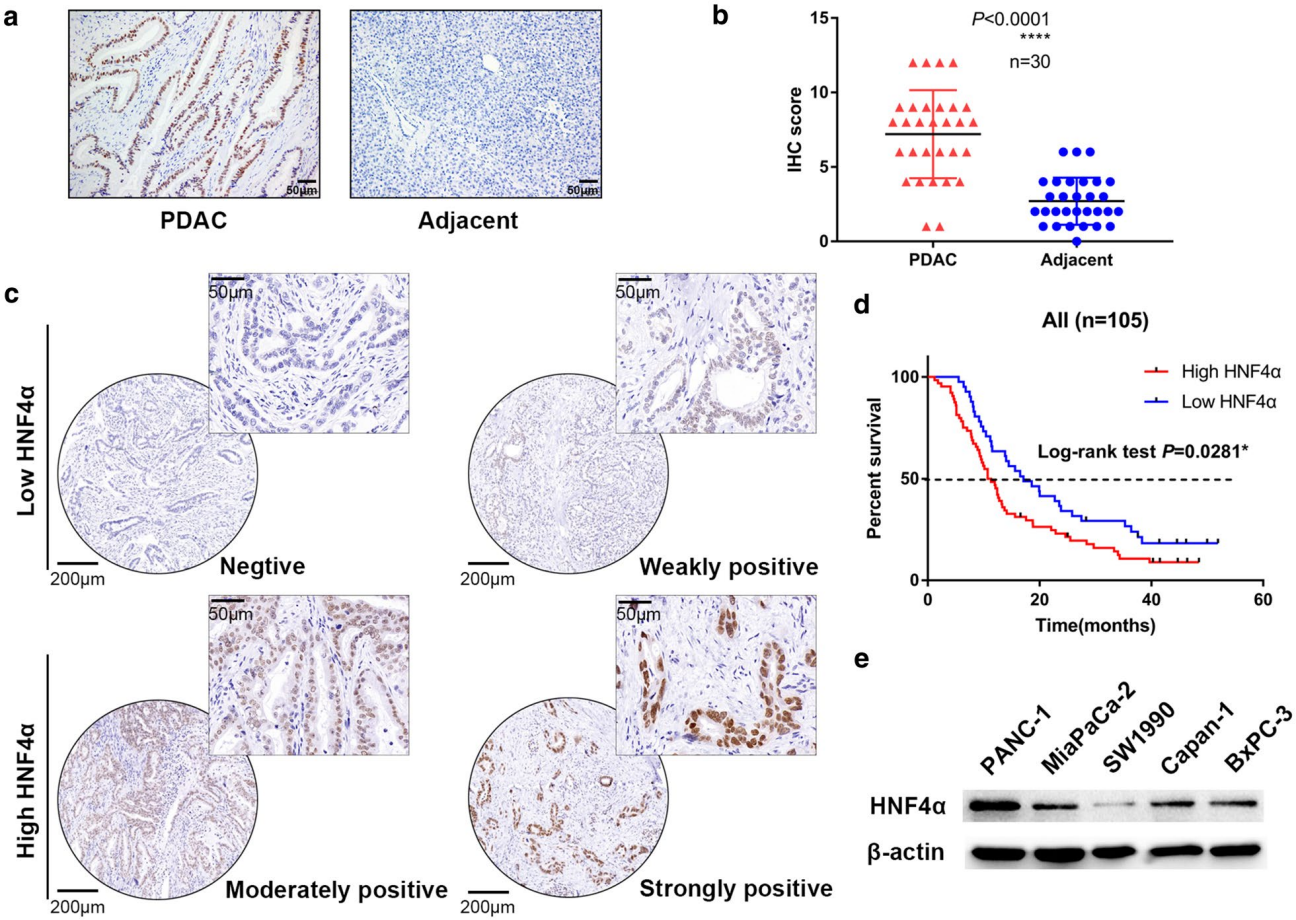
HNF4α expression is increased in PDAC tissues. **a** Representative images of IHC staining for HNF4α in PDAC and adjacent normal tissues (scale bar, 50 μm). **b** HNF4α expression in PDAC and adjacent normal tissues, as determined by the IHC score (n = 30, ****P < 0.0001). **c** Representative images of IHC staining for HNF4α in tissue microarrays (scale bar, 200 µm; inset scale bar, 50 µm). **d** The overall survival of patients with PDAC was analyzed using the Kaplan–Meier analysis on the basis of HNF4α expression (n = 105, *P = 0.0281). **e** Western blotting analysis of HNF4α expression in PDAC cell lines; β-actin was used as a control

We first used IHC to examine the HNF4α levels in 30 paired-patient samples of PDAC and adjacent normal tissues (Fig. 1a, b). Then, we further validated the IHC results by examining HNF4α expression in tissue microarrays containing 105 pairs of patient samples (Fig. 1c, Additional file 1: Table S1). The IHC score showed that HNF4α was expressed at higher levels in PDAC tissues than in control tissues (Additional file 1: Table S1). Moreover, the log-rank test revealed an obvious correlation between high HNF4α expression and poor prognosis of PDAC patients (*P *= 0.0281; Fig. 1d). Pancreatic cancer patients with higher HNF4α expression exhibited significantly shorter overall survival compared to patients with low HNF4α expression, with a median survival time of 11.015 months vs. 17.1 months. Furthermore, according to Cox regression analysis, HNF4α was an independent prognostic marker of PDAC (Table 3). Finally, we investigated the levels of HNF4α protein in various PDAC cell lines (Fig. 1e). HNF4α was highly expressed in PANC-1, Capan-1 and MiaPaCa-2 cell lines. We ultimately chose Capan-1 and MiaPaCa-2 cell lines for further investigation.

**Table 3:** Univariate and multivariate Cox regression of overall survival for patients with PDAC

Characteristics	Univariate			Multivariate		
	HR	95% CI	*P* value	HR	95% CI	*P* value
Age (years)						
> 60	1.195	0.788 to 1.812	0.402		–	
≤ 60						
Gender						
Male	0.842	0.550 to 1.290	0.430		–	
Female						
TNM stage						
IIb, III, IV	1.722	1.133 to 2.619	0.011*		–	
I, IIa						
Tumor size (cm)						
≥ 4.0	1.809	1.148 to 2.851	0.011*	1.919	1.210 to 3.041	0.006**
< 4.0						
Histological grade						
Grade 3	1.200	0.782 to 1.841	0.403		–	
Grade 1,2						
Lymph node status						
Positive	1.826	1.201 to 2.778	0.005**	1.887	1.237 to 1.049	0.003**
Negative						
Vascular emboli						
Positive	1.012	0.571 to 1.791	0.968		–	
Negative						
HNF4α expression						
High	1.560	1.012 to 2.404	0.044*	1.616	1.049 to 2.491	0.030*
Low						

### HNF4α promotes proliferation of pancreatic cancer cells

**Fig. 2:**
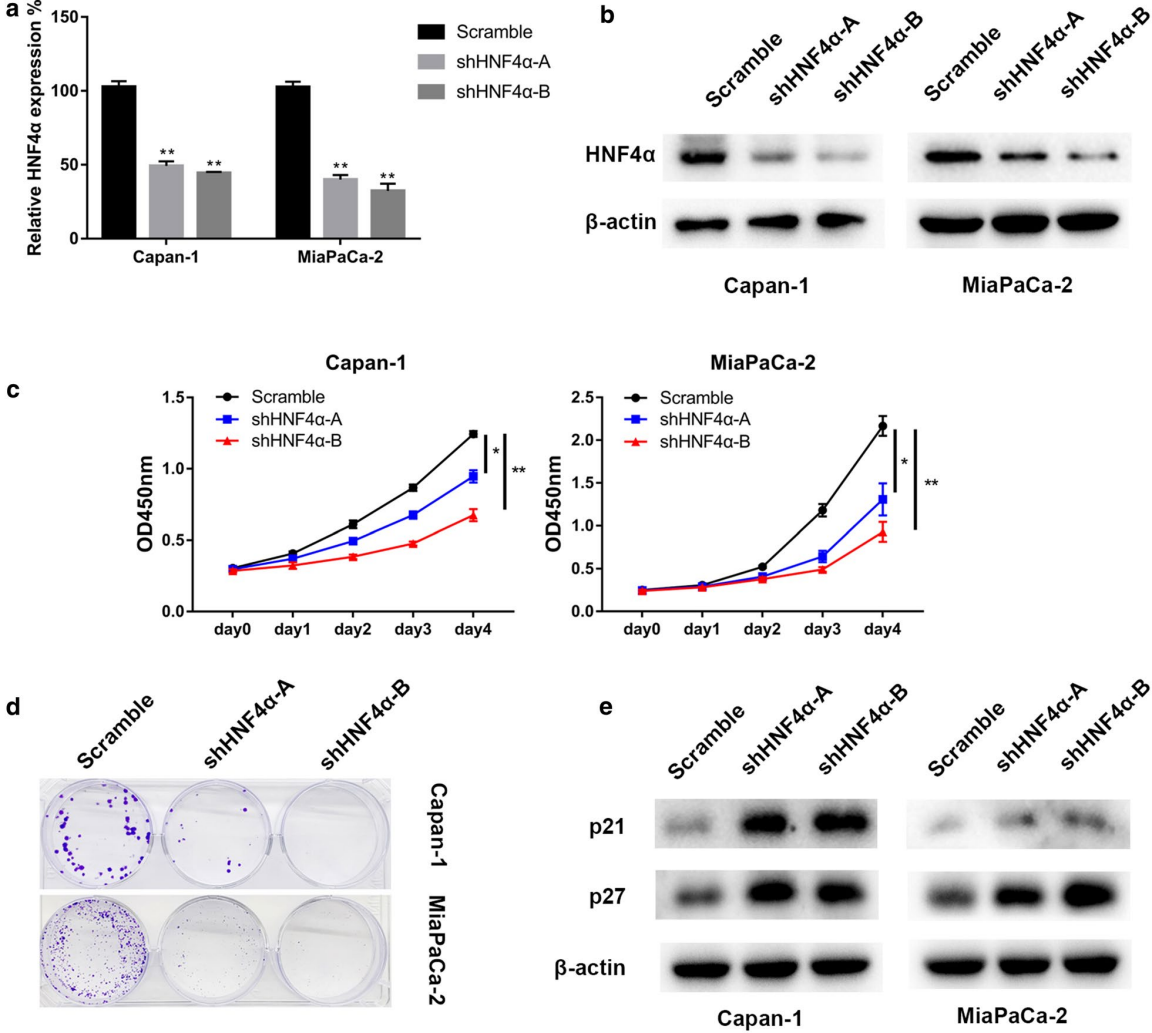
Specific silencing of HNF4α decreased the proliferation of pancreatic cancer cells. **a** Analysis of relative gene expression data of HNF4α using real-time quantitative PCR and the 2-ΔΔCT method. **b** Analysis of protein expression of HNF4α using western blotting assay. **c** CCK-8 assay was used to test the proliferation of PDAC cells transfected with the HNF4α shRNA. **d** Colony formation assay was conducted to confirm the influence of abrogation of HNF4α on pancreatic cancer cell lines; **f** The p21 and p27 expression levels in HNF4α-silencing pancreatic cancer cell lines were analyzed comparing to the controls by western blotting. *Represented significant differences (*P *< 0.05, compared with group control); **Represented with significant differences (*P *< 0.01, compared with group control)

Based on the vital role of HNF4α in PDAC prognosis, we hypothesized that HNF4α might promote pancreatic cancer cell proliferation. Stable HNF4α-silencing cell lines were obtained by infecting Capan-1 and MiaPaCa-2 cells with lentiviral particles and by subsequently using puromycin selection. The knock-down efficiency was verified by qRT-PCR and Western blotting (Fig. 2a, b). The CCK-8 assay showed that HNF4α silencing significantly decreased cell viability in Capan-1 and MiaPaCa-2 cells (Fig. 2c). Subsequent colony formation assay results demonstrated that HNF4α abrogation decreased colony formation capacity in Capan-1 and MiaPaCa-2 cells (Fig. 2d). Further, Western blotting analysis showed increases in p21 and p27 expression in HNF4α-silenced cell lines, which indicated an activated tumor-suppressive mechanism of HNF4α knockdown (Fig. 2e) [27]. In summary, HNF4α may play oncogenic roles and enhances pancreatic cancer cell proliferation.

### Silencing of HNF4α increases pancreatic cancer cell sensitivity to gemcitabine

**Fig. 3:**
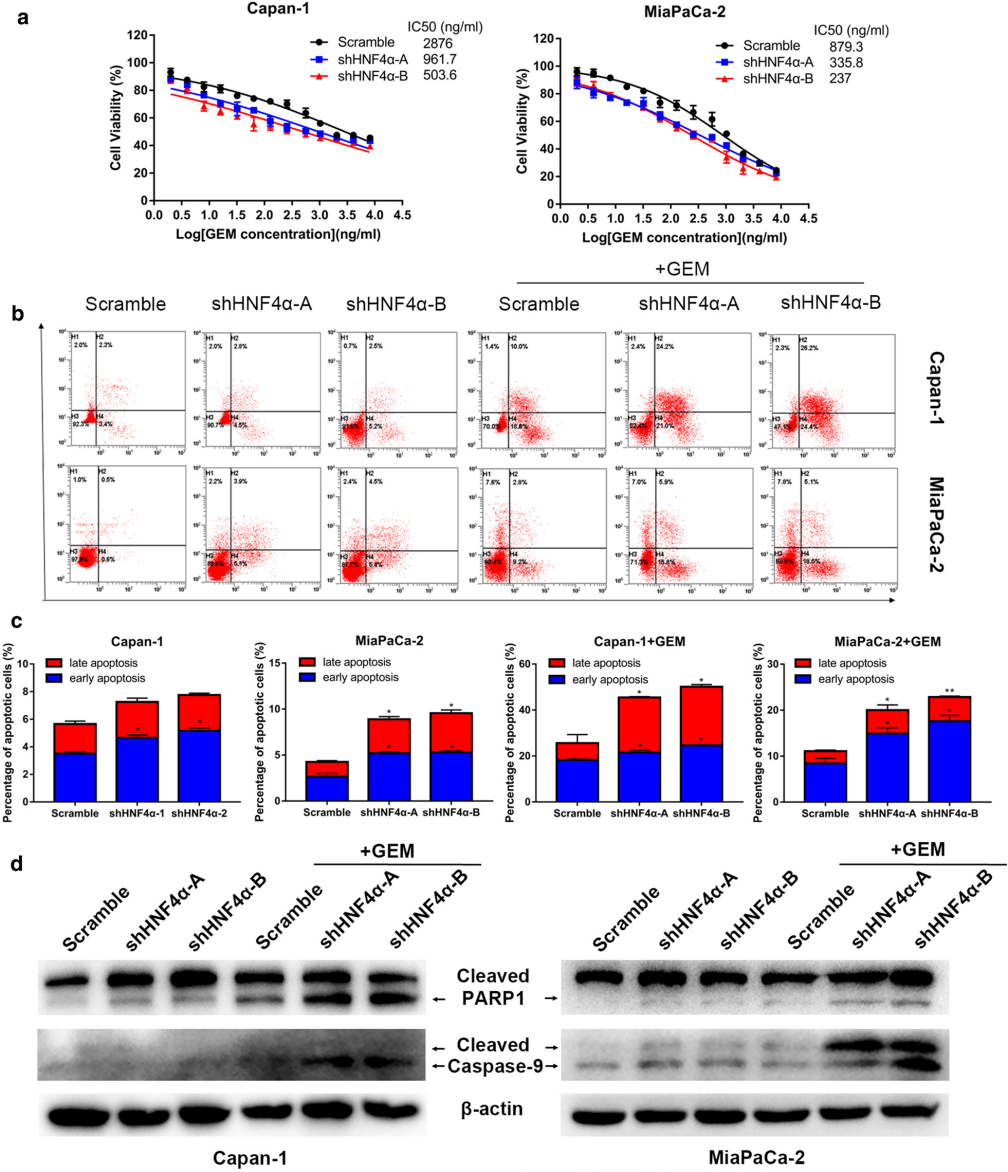
HNF4α knockdown decreases the gemcitabine resistance of pancreatic cancer cells. **a** downregulation of HNF4α decreased IC50 value of gemcitabine in Capan-1 and MiaPaCa-2 cells. **b**, **c** Apoptosis rates of the HNF4α-silenced cell lines with or without gemcitabine treatment. **d** Western blotting analysis of apoptosis-related proteins, cleaved PARP1 and cleaved Caspase-9, of the pancreatic cancer cell lines with or without gemcitabine treatment. *Represented significant differences (*P *< 0.05, compared with group control); **Represented with significant differences (*P *< 0.01, compared with group control)

Since evidence has suggested that loss of p21 or p27 function may contribute to cancer cell chemoresistance [27, 28], we subsequently explored the relationship between HNF4α and gemcitabine sensitivity. We first used a viability assay to analyze the effects of HNF4α abrogation on gemcitabine-treated cells. The results suggested that HNF4α silencing dramatically decreased the IC50 values of gemcitabine both in Capan-1 and MiaPaCa-2 cells at 48 h (Fig. 3a). Gemcitabine exerts its antitumor activity mainly by inducing cell apoptosis [29], and we then investigated gemcitabine-induced apoptosis. The apoptotic rate induced by gemcitabine was further enhanced in HNF4α-silenced cell lines (Fig. 3b, c), with increased expression of apoptosis-related proteins such as cleaved poly (ADP-ribose) polymerase-1 (PARP1) and cleaved caspase-9 (Fig. 3d) [30, 31].

### HNF4α is negatively correlated with hENT1 expression

**Fig. 4:**
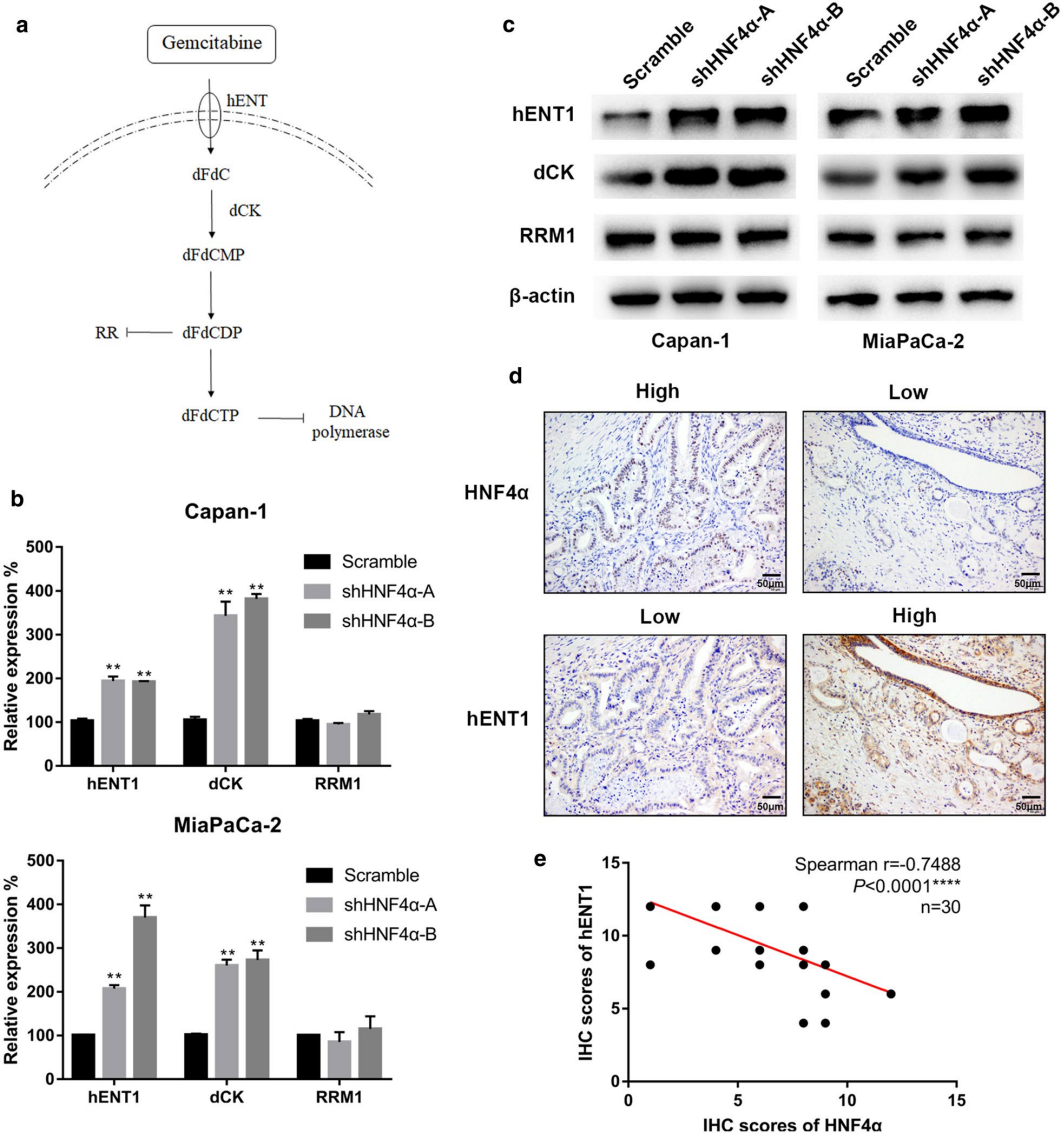
Correlation between the expression of HNF4α and gemcitabine-related proteins. **a** The process of intracellular gemcitabine metabolism. **b** The mRNA levels of hENT1, dCK and RRM1 in pancreatic cancer cell lines were measured by real-time PCR. **c** The protein levels of hENT1, dCK and RRM1 were analyzed by Western blotting following treatment. **d** Representative images of IHC staining for HNF4α and hENT1 in PDAC tissues (scale bar, 50 μm). **e** Correlation analysis of HNF4α expression and hENT1 expression in PDAC tissues, as determined by the IHC score (n = 30, ****P < 0.0001). *Represented significant differences (*P* < 0.05, compared with group control); **Represented with significant differences (*P *< 0.01, compared with group control); *dCK* deoxycytidine kinase, *dFdCMP* gemcitabine monophosphate, *dFdCDP* gemcitabine diphosphate, *dFdCTP* gemcitabine triphosphate, *hENT1* human equilibrative nucleoside transporter 1, *RR* ribonucleotide reductase

We next examined whether HNF4α silencing could regulate the expression of gemcitabine metabolism-related proteins. Numerous studies have indicated that hENT1, dCK and RRM1 are key proteins for gemcitabine resistance in pancreatic cancer (Fig. 4a) [19, 20, 32, 33]. The subsequent qRT-PCR and Western blotting results exhibited upregulated expression of hENT1 and dCK after deletion of HNF4α in Capan-1 and MiaPaCa-2 cell lines, whereas RRM1 presented no obvious change at both the transcriptional and protein levels (Fig. 4b, c). To further validate the results of the in vitro experiments, we examined the correlation between hENT1, dCK and HNF4α in PDAC patients. We found that hENT1 expression negatively correlated with HNF4α levels in PDAC patient tissue samples (Fig. 4d, e). Moreover, consistent with the results of previous studies, the IHC staining of tissue microarrays indicated that higher hENT1 expression indicated a better prognosis in PDAC (Additional file 2: Figure S1, a, b, n = 105, *P *= 0.0208). However, Spearman’s correlation showed no obvious correlation between dCK and HNF4α levels in PDAC patient tissue samples (Additional file 3: Figure S2, n = 30, *P *= 0.4281).

### hENT1 is a downstream target of HNF4α

**Fig. 5:**
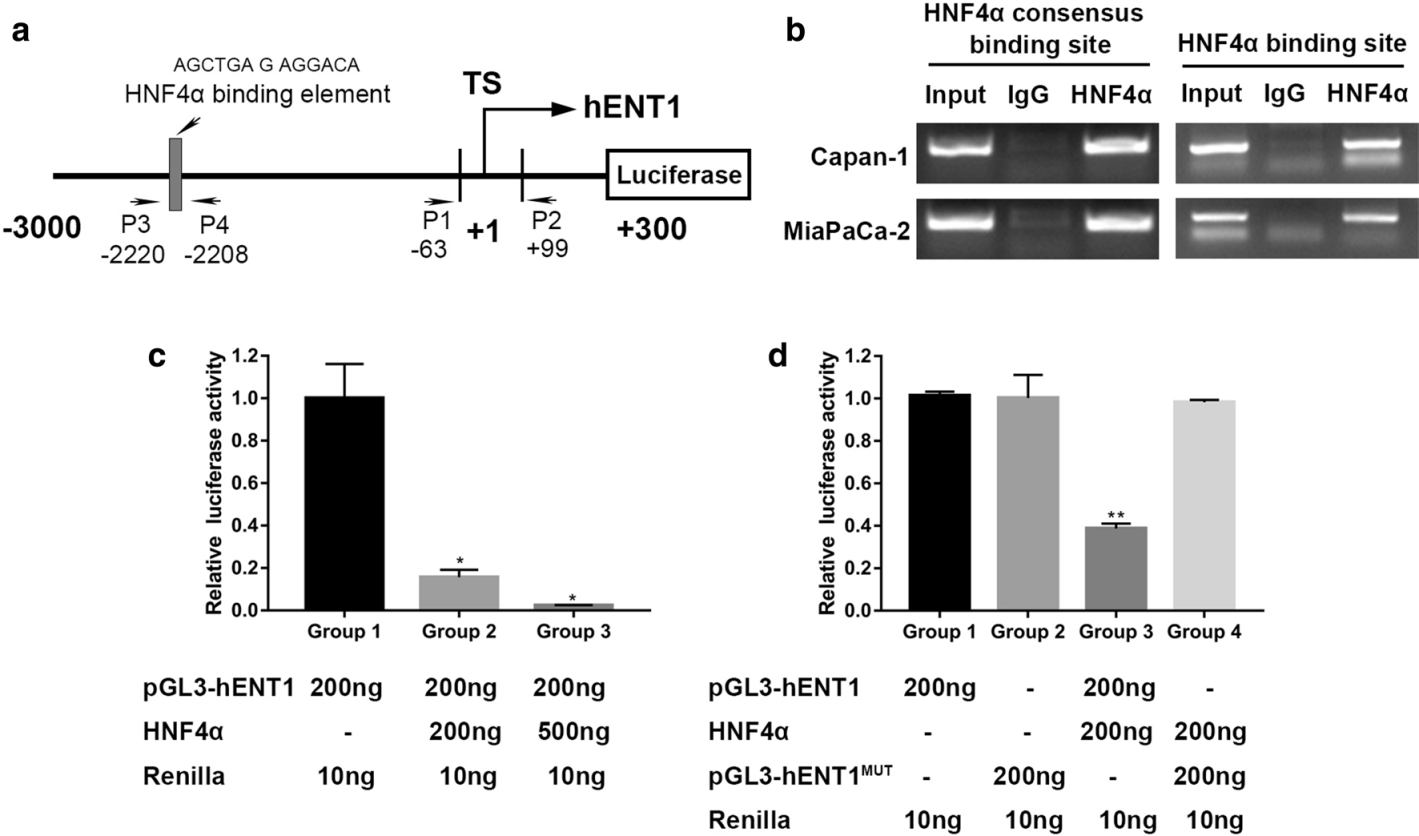
HNF4α was involved in the hENT1 transcriptional expression in PDAC. **a** The position of the HNF4α binding sites in the hENT1 promoter. **b** HNF4α occupies the binding sites of the hENT1 promoter region in Capan-1 and MiaPaCa cells, as measured by ChIP assay. **c** HNF4α affected hENT1 promoter activity in HEK-293T cells. **d** HNF4α didn’t affect the mutated hENT1 promoter activity in HEK-293T cells

Based on these results, we hypothesized that hENT1 may be a target of and regulated by HNF4α. In the promoter region of hENT1, one possible HNF4α binding element exists (Fig. 5a). Thus, we conducted a ChIP assay to determine whether HNF4α could occupy the consensus HNF4α binding element. ChIP results demonstrated that HNF4α could bind the HNF4α binding element in the promoter region of hENT1. Moreover, ChIP results also demonstrated that HNF4α could occupy the region from − 63 to + 99, indicating that other regulatory mechanisms may exist (Fig. 5b). Subsequent luciferase assays demonstrated that manipulation of HNF4α expression inhibited hENT1 promoter activity in a dose-dependent manner (Fig. 5c). This phenomenon was further confirmed by the mutation of hENT1 binding sites; when the sequence was mutated from AGCTGAGAGGACA into AGCTGAGACCAAA to generate a mutant, HNF4α lost its repressive function for the mutated luciferase construct (Fig. 5d).

**Fig. 6:**
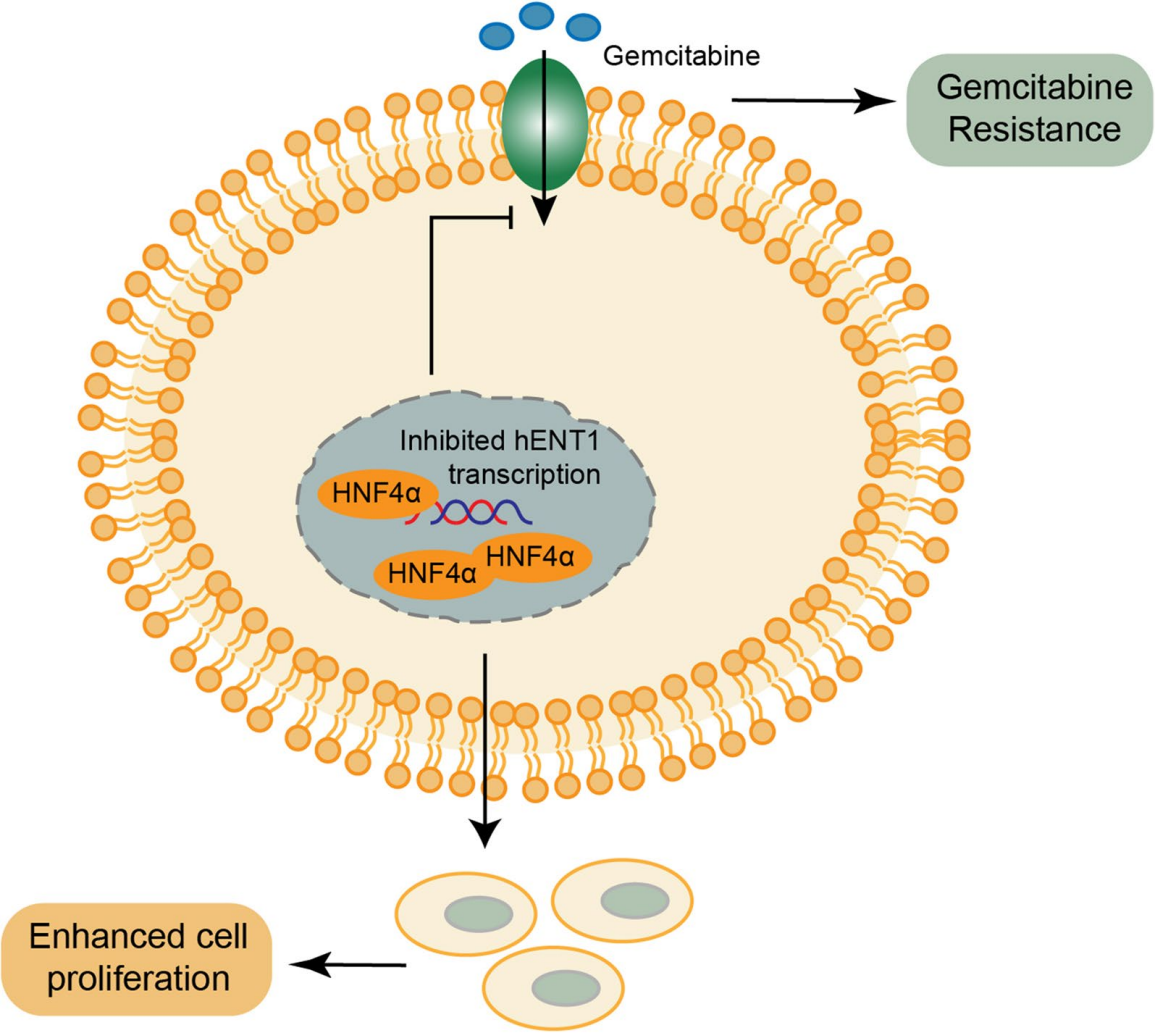
Schematic representation of the model. The model indicates the mechanism of HNF4α-mediated regulation of gemcitabine metabolism via hENT1 in pancreatic cancer cells and the role of HNF4α in cancer cell proliferation

In conclusion, our results demonstrated that HNF4α is a novel predictive marker for overall survival in PDAC. In vitro cell line studies demonstrated that HNF4α promoted proliferation and gemcitabine resistance to PDAC cancer cell lines. Mechanistically, HNF4α suppressed the expression of hENT1, which was responsible for gemcitabine uptake and was correlated with gemcitabine resistance (Fig. 6).

## Discussion

Considering the significant role of gemcitabine in adjuvant therapy and the treatment of patients with unresectable PDAC, elucidating the underlying mechanisms of gemcitabine resistance could improve treatment response [19, 34]. In the present study, we investigated the role of HNF4α in PDAC. Our results indicated that HNF4α enhances pancreatic cancer cell proliferation and promotes gemcitabine resistance by downregulating the transcription of hENT1. Moreover, HNF4α levels constitute an important prognostic factor for patients with PDAC.

HNF4α is a highly conserved nuclear receptor belonging to the nuclear receptor (NR) superfamily [6]. As the third class of nuclear receptors, orphan receptors are characterized by a lack of endogenous ligands [35]. Despite a well-known role in maintaining cellular homeostasis, NRs have also been found to participate in cancer proliferation and progression [36, 37]. NRs play diverse roles in the oncogenesis and progression of many cancers. Estrogen receptor α and androgen receptor are widely recognized as oncogenes, while other NRs, such as proliferator-activated receptor γ, may have inhibitory functions on cancer proliferation [38–40]. Moreover, certain NRs may have opposing functions in different cancer types. For example, the orphan nuclear receptor NR4A1 (Nur77) played a role in suppressing hepatocellular carcinoma by inducing a glycolysis to gluconeogenesis switch, whereas it facilitated cancer cell proliferation via the ROS/endoplasmic reticulum stress pathways in pancreatic cancer [41, 42]. NRs also play an important role in regulating drug metabolism in cancer therapy. Holbeck et al. reported that cancer sensitivity to microtubule-disrupting drugs, such as derivatives of vinblastine, colchicines, and Taxol, was increased in cells expressing low levels of NR2F2 based on NR expression profiling, while the high levels of the orphan receptor tailless were correlated with 9α-fluoroprednisolone sensitivity [43].

Considering the significant roles of NRs in cancer development and the high expression of HNF4α in gastrointestinal cancers, such as colorectal carcinoma [43], we investigated whether HNF4α regulated the biological behaviors of pancreatic cancer. We found that abrogation of HNF4α expression inhibited pancreatic cancer cell proliferation and induced cell apoptosis, with increased expression of the cyclin-dependent protein kinase inhibitors p21 and p27. HNF4α has been reported to inhibit hepatocyte proliferation via several proposed mechanisms, including direct inhibition of mitogenic genes, regulation of the c-Myc and cyclin pathways, and an HNF4α-driven miRNA feedback loop [36]. Our results indicated that HNF4α may promote cell proliferation via the cyclin pathways in pancreatic cancer, but further investigation is needed to confirm this hypothesis. Moreover, in vivo experiments are required to support the oncogenic role of HNF4α in PDAC in the future.

The mechanisms of chemoresistance of PDAC may involve the hypoxic tumor microenvironment and deficient vascularization, remodeled metabolism, the capacity for epithelial-mesenchymal transition and altered key signaling pathways, such as the PI3 K/Akt and NF-κB signaling pathways [19, 44–49]. As a key transporter for the cellular uptake of gemcitabine, the downregulation of hENT1 is one of the recognized mechanisms of gemcitabine resistance [19]. Although the role of hENT1 as a predictive molecule for the response to gemcitabine-based therapy has been confirmed by many studies using adjuvant treatment and studying patients with unresectable tumors, in which higher expression of hENT1 was correlated with better survival, the molecular mechanisms of hENT1 regulation have seldom been described [20, 50–52]. Pandolfi et al. reported that high levels of glucose could upregulate the formation of hCHOP-C/EBPa, a complex that represses hENT1 expression [53]. A. Hesler et al. found that hENT1 could be negatively regulated by the matricellular protein cysteine-rich angiogenic inducer 61, but the underlying mechanism requires further exploration [54]. Hu et al. proposed the role of FBW7 in the regulation of hENT1 at the protein level, possibly via the inhibition of the lysosome degradation by hENT1 [24].

Our study revealed that hENT1 could be regulated by HNF4α at the transcriptional level. HNF4α mainly binds to a 6-bp repeat (AGGTCA) with a nucleotide spacer called direct repeat 1 (DR1) [55, 56]. Our results showed that HNF4α recognized the consensus binding site located on the promoter region of hENT1 and activated downstream transcription. Moreover, a relatively nonconserved region with abundant CpG dinucleotide could also be recognized by HNF4α, which indicates that HNF4α might participate in the methylation process of hENT1. A previous study demonstrated DNA methylation-independent hENT1 downregulation, which correlated with acquired gemcitabine resistance in cancer cells [57]. Our study supports the need for further investigation of the underlying mechanism of methylation-dependent hENT1 regulation. Moreover, after binding to DNA in the form of a homologous dimer, how HNF4α recruits transcriptional corepressors and accessory proteins to regulate hENT1 is unclear. HNF4α has been reported by previous studies to interact with chromatin modifiers, such as PRMT1 and BMI1 [58, 59], suggesting a potential role of HNF4α in chromatin methylation. Furthermore, in addition to the expression level of hENT1, hENT1 transporter activity is another important factor that affecting the uptake of nucleoside analogs. A study has reported that although the mRNA level of hENT1 in chronic lymphocytic leukemia cells is increased with the presence of interleukin 4 (IL-4), the hENT1-dependent uridine uptake remained unchanged [60]. Another study using a murine model of chronic myelogenous leukemia showed a decreased ENT1 activity reduction following reduced mRNA level of ENT1 [61]. Therefore, the transcription of hENT1 may not perfectly correlated with the uridine uptake activity, dual targeting hENT1 transcription and transporter activity might provide the utmost efficacy in gemcitabine utilization. Thus, a protein interaction screening strategy is needed to determine whether HNF4α is involved in epigenetic regulation or posttranslational modification of hENT1.

## Conclusions

In conclusion, we demonstrated that HNF4α functioned as a tumor promoter gene that was upregulated in pancreatic cancer. Moreover, we discovered that higher expression of HNF4α is correlated with poorer prognosis in PDAC patients. HNF4α promotes pancreatic cancer cell proliferation and reduces gemcitabine-induced cell apoptosis. Mechanistically, we found that HNF4α could downregulate hENT1, a membrane transporter of gemcitabine, at the transcriptional level. Taken together, these findings suggest that HNF4α may serve as a potential novel predictive and therapeutic target for pancreatic cancer.

## Additional files

**Additional file 1: Table S1.** HNF4α expression between PDAC and adjacent tissue in tumor tissue microarray.

**Additional file 2: Figure S1.** hENT1 expression is a prognostic factor in PDAC. **a** Representative images of IHC staining for hENT1 in tissue microarrays (scale bar, 200 µm; inset scale bar, 50 µm). **b** The overall survival of patients with PDAC was analyzed using the Kaplan–Meier analysis on the basis of hENT1 expression (n = 105, *P = 0.0208).

**Additional file 3: Figure S2.** Correlation between the expression of HNF4α and dCK. Correlation analysis of HNF4α expression and dCK expression in PDAC tissues, as determined by the IHC score (n = 30, *P* = 0.4281).

## Abbreviations

Bcl-2: B cell lymphoma 2
ChIP: chromatin immunoprecipitation
dCK: deoxycytidine kinase
DR1: direct repeat 1
FBS: foetal bovine serum
hENT1: human equilibrative nucleoside transporter 1
HNF4α: hepatocyte nuclear factor 4α
IHC: immunohistochemical
IL-4: interleukin 4
NR: nuclear receptor
PDAC: pancreatic adenocarcinoma
PARP1: poly ADP-ribose polymerase-1
qRT-PCR: quantitative real-time PCR
RR: ribonucleotide reductase
TNM: tumor node metastasis
