# Supplementary material for: Role of hepatocyte nuclear factor 4 alpha in cell proliferation and gemcitabine resistance in pancreatic adenocarcinoma
Source: Cancer Cell Int. 2019 Mar 4;19:49. doi: 10.1186/s12935-019-0767-4 (PMC6398265; doi:10.1186/s12935-019-0767-4)
Supplement: Supplementary file 1 — Additional file 1: Table S1. HNF4α expression between PDAC and adjacent tissue in tumor tissue microarray. [file 12935_2019_767_MOESM1_ESM.docx]

**Supplementary Table S1.**

**HNF4α expression between PDAC and adjacent tissue in tumor tissue microarray.**

| HNF4α expression | HNF4α expression in tumor tissue of pancreatic cancer | | |
| --- | --- | --- | --- |
|  | PDAC | Adjacent tissue | *P* value |
|  |  |  | <0.0001 |
| High | 64 | 29 |  |
| Low | 41 | 76 |  |
