# Supplementary figures and images for: Role of hepatocyte nuclear factor 4 alpha in cell proliferation and gemcitabine resistance in pancreatic adenocarcinoma
Source: Cancer Cell Int. 2019 Mar 4;19:49. doi: 10.1186/s12935-019-0767-4 (PMC6398265; doi:10.1186/s12935-019-0767-4)

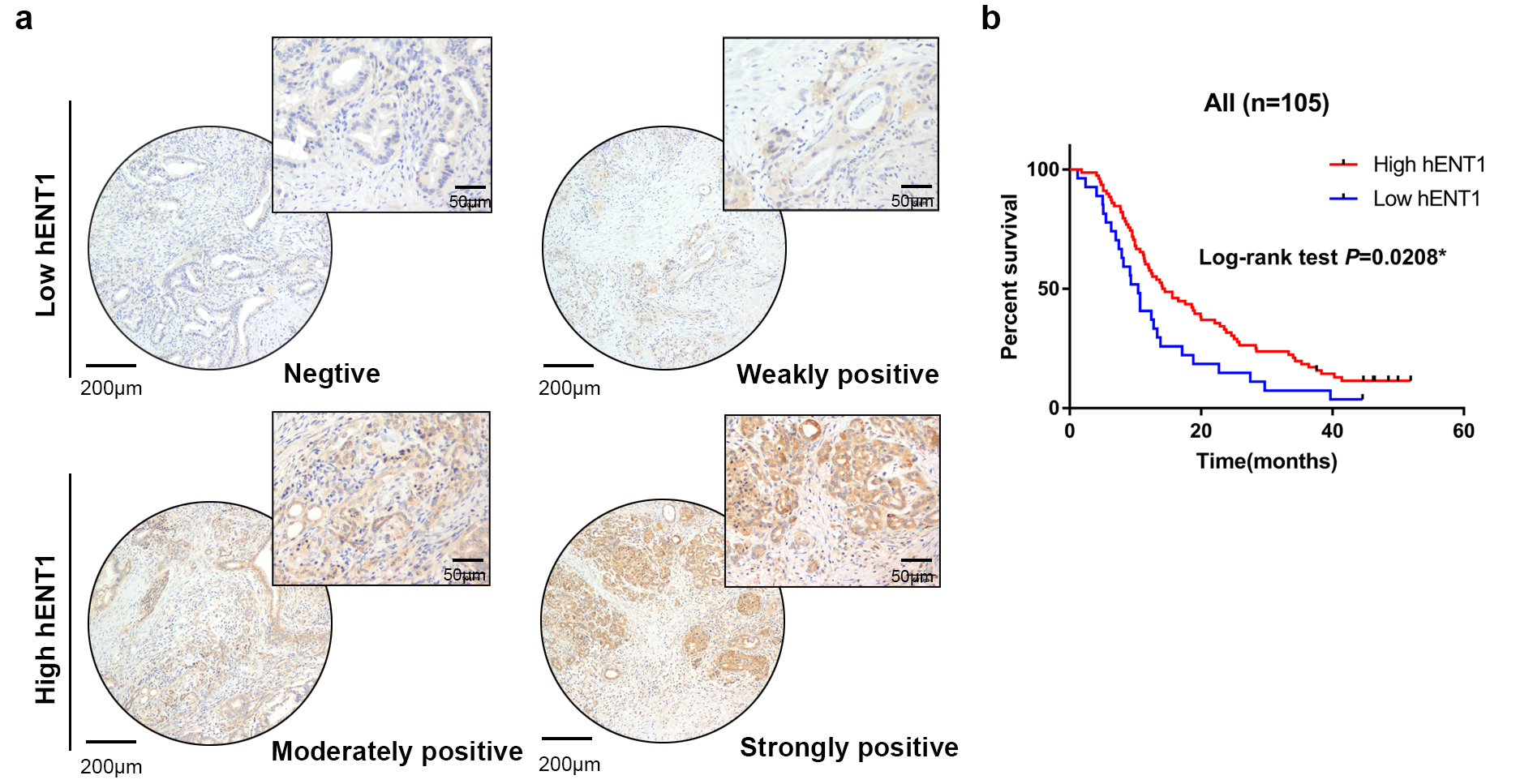

Supplement: Supplementary file 2 — Additional file 2: Figure S1. hENT1 expression is a prognostic factor in PDAC. a Representative images of IHC staining for hENT1 in tissue microarrays (scale bar, 200 µm; inset scale bar, 50 µm). b The overall survival of patients with PDAC was analyzed using the Kaplan–Meier analysis on the basis of hENT1 expression (n = 105, *P = 0.0208). [file 12935_2019_767_MOESM2_ESM.jpg]

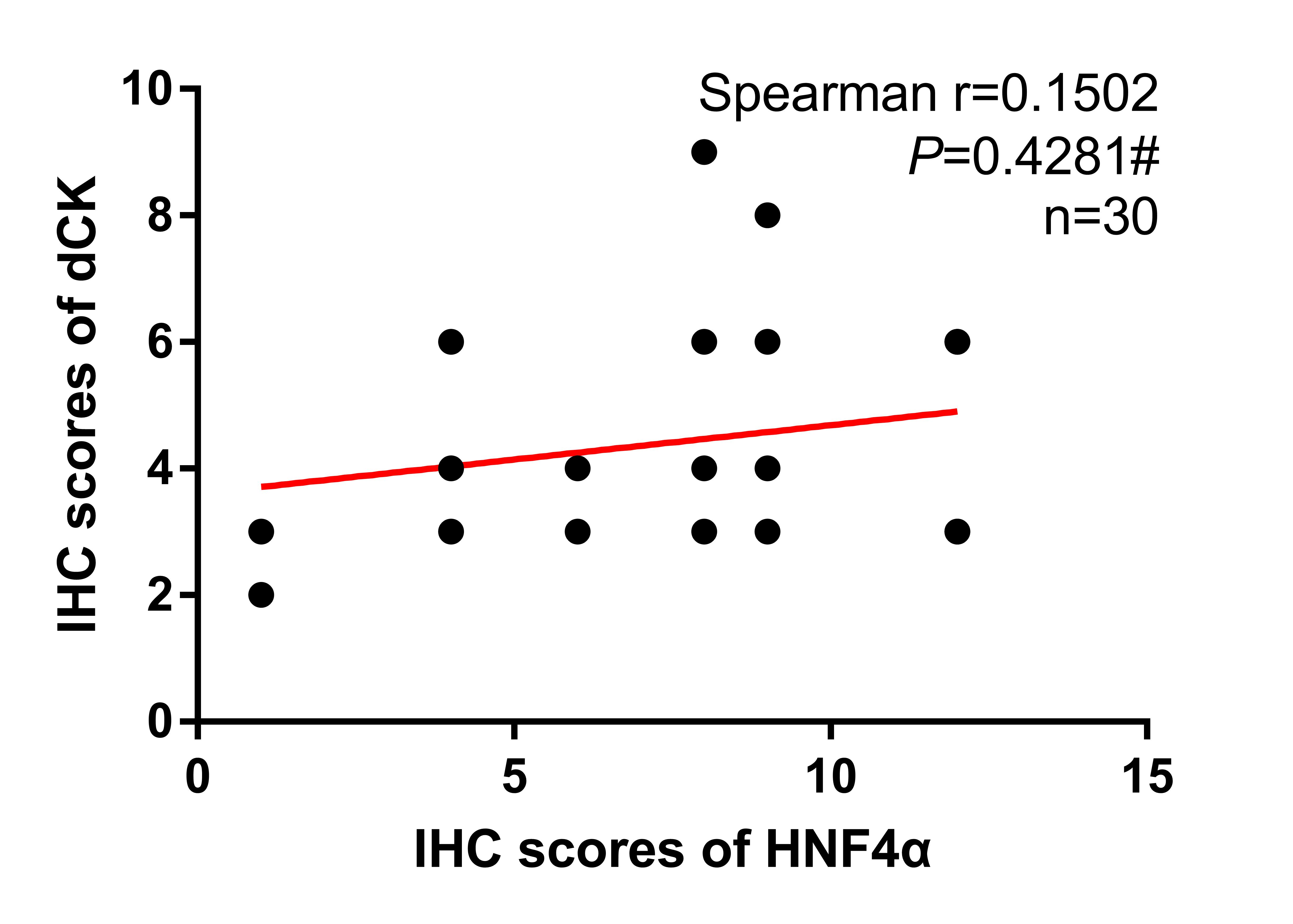

Supplement: Supplementary file 3 — Additional file 3: Figure S2. Correlation between the expression of HNF4α and dCK. Correlation analysis of HNF4α expression and dCK expression in PDAC tissues, as determined by the IHC score (n = 30, P = 0.4281). [file 12935_2019_767_MOESM3_ESM.jpg]
